# Characterization of a Novel Laccase LAC-Yang1 from White-Rot Fungus *Pleurotus ostreatus* Strain Yang1 with a Strong Ability to Degrade and Detoxify Chlorophenols

**DOI:** 10.3390/molecules26020473

**Published:** 2021-01-18

**Authors:** Xinping Liu, Wei Deng, Yang Yang

**Affiliations:** Hubei Key Laboratory of Genetic Regulation and Integrative Biology, School of Life Sciences, Central China Normal University, Wuhan 430079, China; lxp921113@163.com (X.L.); dengw112@163.com (W.D.)

**Keywords:** fungi, laccase, enzymatic properties, chlorophenol, degradation, detoxification

## Abstract

In this study, a laccase LAC-Yang1 was successfully purified from a white-rot fungus strain *Pleurotus ostreatus* strain yang1 with high laccase activity. The enzymatic properties of LAC-Yang1 and its ability to degrade and detoxify chlorophenols such as 2,6-dichlorophenol and 2,3,6-trichlorophenol were systematically studied. LAC-Yang1 showed a strong tolerance to extremely acidic conditions and strong stability under strong alkaline conditions (pH 9–12). LAC-Yang1 also exhibited a strong tolerance to different inhibitors (EDTA, SDS), metal ions (Mn^2+^, Cu^2+^, Mg^2+^, Na^+^, K^+^, Zn^2+^, Al^3+^, Co^2+^, and metal ion mixtures), and organic solvents (glycerol, propylene glycol). LAC-Yang1 showed good stability in the presence of Mg^2+^, Mn^2+^, glycerol, and ethylene glycol. Our results reveal the strong degradation ability of this laccase for high concentrations of chlorophenols (especially 2,6-dichlorophenol) and chlorophenol mixtures (2,6-dichlorophenol + 2,3,6-trichlorophenol). LAC-Yang1 displayed a strong tolerance toward a variety of metal ions (Na^2+^, Zn^2+^, Mn^2+^, Mg^2+^, K^+^ and metal ion mixtures) and organic solvents (glycerol, ethylene glycol) in its degradation of 2,6-dichlorophenol and 2,3,6-trichlorophenol. The phytotoxicity of 2,6-dichlorophenol treated by LAC-Yang1 was significantly reduced or eliminated. LAC-Yang1 demonstrated a good detoxification effect on 2,6-dichlorophenol while degrading this compound. In conclusion, LAC-Yang1 purified from *Pleurotus ostreatus* has great application value and potential in environmental biotechnology, especially the efficient degradation and detoxification of chlorophenols.

## 1. Introduction

White-rot fungus is a class of filamentous fungi that forms white decay on wood. With the strong ability of lignin decomposition, white-rot fungus has good potential in numerous energy and environmental biotechnology applications, including the development and utilization of renewable resources, and the biodegradation of different xenobiotic compounds [1,2]. *Pleurotus ostreatus* is a type of edible fungus with great edible and nutritional value. Recently, this fungus has also been studied as source of bioactive proteins, such as a specific ribonuclease (ribotoxin-like protein) able to inhibit protein synthesis in vitro. A novel ribotoxin-like protein named Ostreatin was purified and characterized from *P. ostreatus* [3]. *P. ostreatus* is also an important member of white-rot fungus. *P. ostreatus* and its ligninolytic enzymes also have important application values in the field of the treatment of environmental pollutants and bioremediation [4].

Laccase (EC 1.10.3.2) is a type of polyphenol oxidase with four copper atoms in its catalytic site. Laccase catalyzes the oxidation of phenols and aromatic compounds, and by transferring four electrons, reduces molecular oxygen to water. The four copper atoms in the active site of laccase combine with oxygen to form a copper superoxide complex and an electron transport chain for redox reactions [5,6,7]. In recent years, laccase has demonstrated good performance in the degradation of industrial dyes [8], polycyclic aromatic hydrocarbons [9], endocrine disruptors such as bisphenol A [10,11], pesticides [12], mycotoxin [13], antibiotics [14,15], diclofenac [16] and other low-degradability organic pollutants.

Chlorophenols are chlorine-containing aromatic compounds and are widely used in present-day industry as important raw chemical materials. Because of their toxicity, potential carcinogenic and mutagenic effects, and low degradability, which make them persistent organic pollutants, the widespread application of chlorophenol compounds in industrial production and the discharge of industrial wastewater containing these compounds have brought serious environmental pollution problems. Study on the degradation of chlorophenols is thus of importance and value to the management of the ecological environment and human health [17,18,19]. Laccase has been shown to yield good degradation results for many chlorophenols, such as 2-chlorophenol, 2,4-dichlorophenol, 2,4,6-trichlorophenol, and pentachlorophenol [20,21,22,23,24,25]. The degradation ability of laccase varies with the source of the laccase. The degradation of chlorophenol by laccase also depends on the number of chlorine atoms and their position in the phenol structure [26]. Most of the research on laccase degradation of chlorophenol focuses on the degradation of a single type of chlorophenol, and few studies have been performed on the degradation of chlorophenol mixtures [27,28]. The immobilized laccase has been applied to effectively remove phenolic compounds such as phenol, 4-chlorophenol, 2,4-dichlorophenol and 2,4,6-trichlorophenol [29,30].

Although some researchers have previously studied the degradation of different chlorophenols by laccase, some key problems remain to be explored and solved to achieve better application of this enzyme and more efficient degradation of chlorophenols: (1) in an actual polluted environment, chlorophenols are largely found in the industrial waste and industrial wastewater discharge, and other metal ions and organic solvents are usually present at high concentration in the chlorophenol pollutants encountered in real life [17]. What are the effects of metal ions and organic solvents on the degradation of chlorophenols by laccase? (2) Could laccase also achieve a good detoxification effect while degrading chlorophenols? These questions deserve further exploration. It is of great scientific importance and practical value to search for laccase that shows a high tolerance to a wide array of organic solvents and metal ions and to investigate its detoxification of chlorophenols. In this way, the laccase can be more effectively utilized in the degradation of chlorophenols in real-life polluted environments.

In this study, a laccase LAC-Yang1 was isolated and purified from a white-rot fungus strain *P. ostreatus* strain yang1 with high laccase activity. The enzymatic properties of this laccase and its ability to degrade chlorophenols with different chemical structures (such as 2,6-dichlorophenol, 2,3,6-trichlorophenol, and chlorophenol mixtures) were systematically studied. The tolerance of LAC-Yang1 toward different organic solvents and metal ions in the degradation of 2,6-dichlorophenol (2,6-DCP) and 2,3,6-trichlorophenol (2,3,6-TCP) was emphasized. Detoxification of chlorophenol by LAC-Yang1 was also investigated. LAC-Yang1 has great application value and potential in the degradation and detoxification of chlorophenols.

## 2. Results

### 2.1. Purification of Laccase LAC-Yang1 from P. ostreatus Strain Yang1

**Figure 1:**
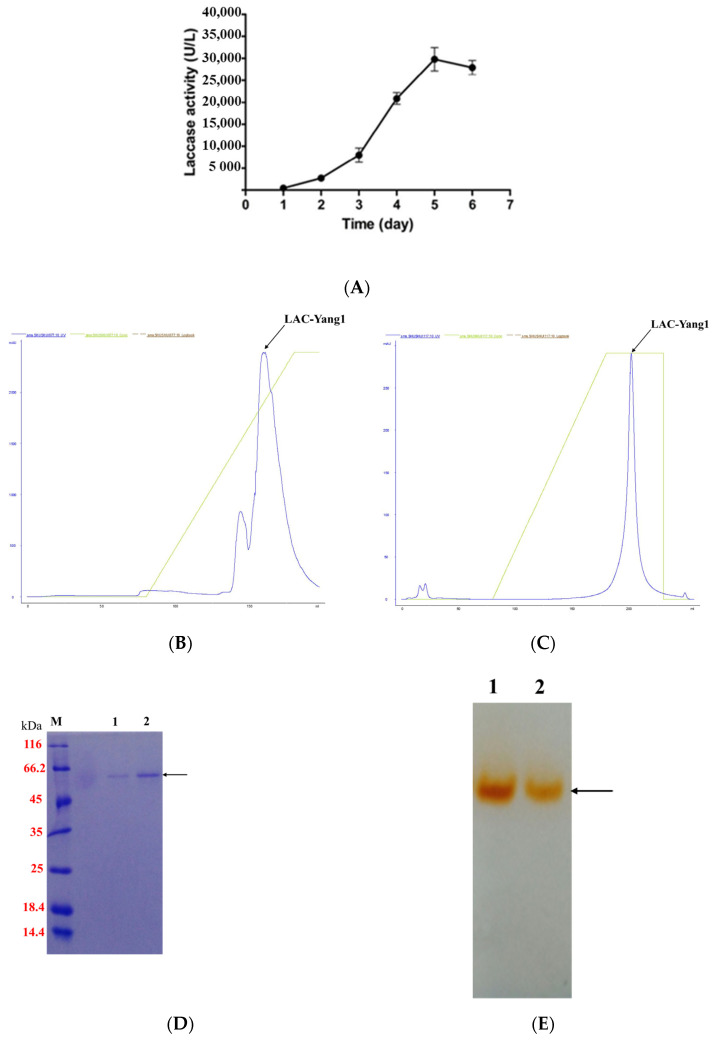
Purification of LAC-Yang1 from *P. ostreatus* strain yang1. (**A**) The laccase production of *P. ostreatus* strain yang1 after the addition of syringic acid and Cu^2+^. (**B**) The chromatographic profile of LAC-Yang1 (anion exchange chromatography). (**C**) The chromatographic profile of LAC-Yang1 (hydrophobic chromatography). (**D**) SDS-PAGE analysis of LAC-Yang1. Lane M: protein molecular mass marker. Lane 1–2: purified LAC-Yang1. (**E**) Native-PAGE analysis of LAC-Yang1. Lane 1–2: purified LAC-Yang1.

Cu^2+^ and syringic acid were used as inducers to induce laccase production by the fungal strain in tomato juice medium. As shown in Figure 1A, laccase activity began to rise rapidly three days after inducer addition, reaching its highest level of 29,753.09 U/L five days after inducer addition. As shown in Figure S1 (Supplementary Materials), only one laccase was secreted by *P. ostreatus* strain yang1. LAC-Yang1 was purified by precipitation with ammonium sulfate, anion exchange chromatography and hydrophobic interaction chromatography. The summary of the purification process is shown in Table 1. The chromatographic profiles of the purified LAC-Yang1 are shown in Figure 1B,C.

The purified LAC-Yang1 showed only one band in SDS-PAGE and Native-PAGE (Figure 1D,E). The molecular weight of LAC-Yang1 was approximately 55.6 kDa. The specific activity of purified LAC-Yang1 was 63.64 U/mg.

### 2.2. Enzymatic Properties of Purified LAC-Yang1

#### 2.2.1. Kinetic Studies on the Purified LAC-Yang1

The constants of enzyme-catalyzed reactions and reaction kinetics of purified LAC-Yang1 were determined using ABTS, 2,6-DMP, and guaiacol as substrates. As shown in Table 2, the maximum rate of LAC-Yang1-catalyzed reactions was observed with ABTS as a substrate (V_max_ = 4.41 × 10^−7^ mM/s), and the lowest reaction rate was observed on guaiacol substrate. The affinity of LAC-Yang1 for the three substrates was 2,6-DMP > ABTS > guaiacol. The conversion efficiency of LAC-Yang1 was higher on ABTS than on the other two substrates (Table 2).

#### 2.2.2. Effect of Temperature on the Activity and Stability of LAC-Yang1

**Figure 2:**
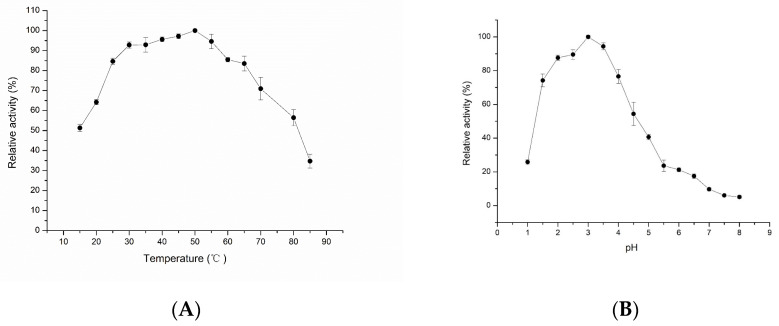
Effect of pH and temperature on the activity of purified LAC-Yang1. (**A**) Effect of temperature on the laccase activity of LAC-Yang1. The laccase activity measured at the optimal temperature was set as 100%. (**B**) Effect of pH on the laccase activity of LAC-Yang1. The laccase activity measured at the optimal pH was set as 100%.

The optimal temperature and stability of LAC-Yang1 were studied. As shown in Figure 2A, the optimal temperature of LAC-Yang1 was 50 °C. LAC-Yang1 still had catalytic activity at a high temperature (Figure 2A).

The stability of LAC-Yang1 at different temperatures is shown in Table 3. The values of t_1/2_ suggested that the activity of LAC-Yang1 remained rather stable at 30 °C and 40 °C. As the temperature increased, the stability of LAC-Yang1 decreased (Table 3).

#### 2.2.3. Effect of pH on the Activity and Stability of LAC-Yang1

The optimal pH and stability of LAC-Yang1 under different pH values were studied. As shown in Figure 2B, the optimal pH of the enzyme was 3.0. The results indicated higher activity of LAC-Yang1 under acid conditions and decreases in its activity with increases of pH to the pH range of 5–8 (Figure 2B).

The stability of LAC-Yang1 at different pH values is shown in Table 3 and Table 4. The enzyme was very stable at pH 9–11 and displayed activity levels of 71.57%, 67.58%, 64.50% after incubation for 7 days at pH 9, 10, and 11, respectively. LAC-Yang1 was most stable at pH 9.0. The above results suggest a good stability of LAC-Yang1 under strong alkaline conditions. However, as the pH decreased, the stability of the enzyme declined significantly and was rather poor under acidic conditions (Table 4).

#### 2.2.4. Effect of Inhibitors on the Activity of LAC-Yang1

The influence of five enzyme inhibitors on the activity of LAC-Yang1 was investigated. As shown in Table 5, LAC-Yang1 demonstrated a strong tolerance to SDS and EDTA-2Na. However, DTT, sodium azide, and mercaptoethanol had a greater impact on LAC-Yang1 activity. DTT and sodium azide induced a significant reduction in enzyme activity at only 0.05 mmol/L (Table 5).

#### 2.2.5. Effect of Metal Ions and Organic Solvents on the Activity of LAC-Yang1

**Figure 3:**
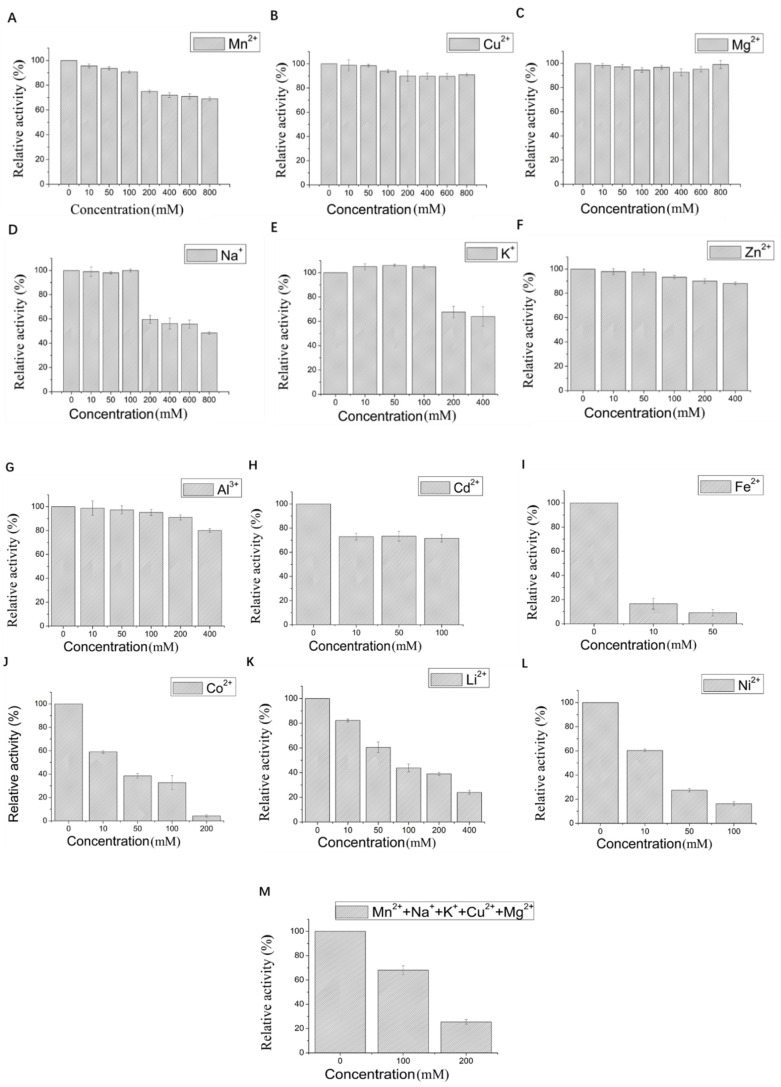

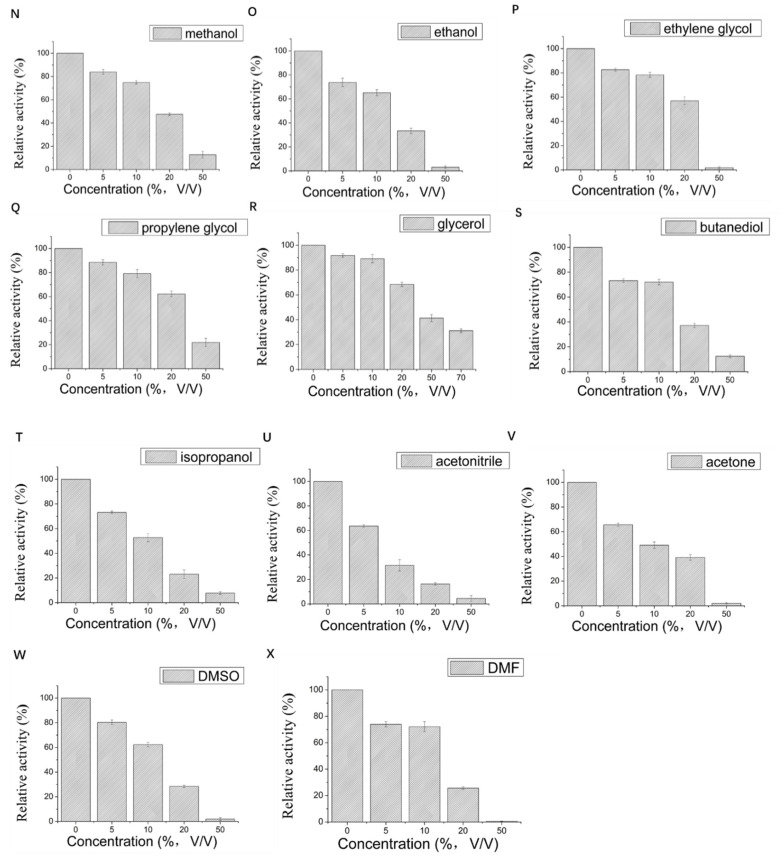
Effect of different metal ions and organic solvents on the laccase activity of LAC-Yang1. (**A**): Mn^2+^, (**B**): Cu^2+^, (**C**): Mg^2+^, (**D**): Na^+^, (**E**): K^+^, (**F**): Zn^2+^, (**G**): Al^3+^, (**H**): Cd^2+^, (**I**): Fe^2+^, (**J**): Co^2+^, (**K**): Li^+^, (**L**): Ni^2+^, (**M**): mixture of five metal ions (Mn^2+^ + Na^+^ + K^+^ + Cu^2+^ + Mg^2+^), (**N**): methanol, (**O**): ethanol, (**P**): ethylene glycol, (**Q**): propylene glycol, (**R**): glycerol, (**S**): butanediol, (**T**): isopropanol, (**U**): acetonitrile, (**V**): acetone, (**W**): DMSO, (**X**): DMF. The laccase activity of the control without adding any metal compound or organic solvent was set as 100%.

The effect of 12 metal ions with different concentrations on the activity of LAC-Yang1 was studied. As shown in Figure 3A–H, LAC-Yang1 showed strong tolerance to various metal ions such as Mn^2+^, Cu^2+^, Mg^2+^, Na^+^, K^+^, Zn^2+^, Al^3+^, Cd^2+^. LAC-Yang1 also had strong tolerance to higher concentrations of Mg^2+^, Cu^2+^ and Mn^2+^ (800 mM) (Figure 3A–C). Among the 12 metal ions, Fe^2+^ inhibited LAC-Yang1 activity. The enzyme activity was reduced to 16.54% by 10 mM concentration of Fe^2+^ (Figure 3I). In addition, LAC-Yang1 also showed tolerance to metal ion mixtures (Mn^2+^ + Na^+^ + K^+^ + Cu^2+^ + Mg^2+^) (Figure 3M). The above results suggested that LAC-Yang1 was not only tolerant to single metal ions but also to mixtures of multiple metal ions.

The effect of 11 organic solvents with different concentrations on the activity of LAC-Yang1 was also studied. As shown in Figure 3N–X, among the 11 organic solvents tested, LAC-Yang1 showed greater tolerance to glycerol and propylene glycol (Figure 3R,Q). Even at 70% concentration of glycerol, the activity of LAC-Yang1 could still reach 31.20% (Figure 3R). Acetonitrile, isopropanol and DMF had a strong inhibitory effect on LAC-Yang1 (Figure 3T,U,X). The above results indicated that LAC-Yang1 was most tolerant to glycerol and most sensitive to acetonitrile.

#### 2.2.6. Effect of Metal Ions and Organic Solvents on the Stability of LAC-Yang1

The effect of metal ions with different concentrations on the stability of LAC-Yang1 was studied (Table 6). As shown in Table 6, t_1/2_ of Mg^2+^ (2 mM) and Mg^2+^ (5 mM) was 24 h and 12 h, respectively. The values of t_1/2_ suggest that LAC-Yang1 was more stable in the presence of Mg^2+^ and Mn^2+^.

The effect of organic solvents with different concentrations on the stability of LAC-Yang1 was also studied (Table 7). The values of t_1/2_ suggest that LAC-Yang1 showed relatively strong stability in the presence of glycerol and ethylene glycol. t_1/2_ of glycerol (5%, *v*/*v*) and glycerol (10%, *v*/*v*) was 72 h and 24 h, respectively. Among the 11 organic solvents studied, LAC-Yang1 was most stable in the presence of glycerol (Table 7). After incubation with 50% concentration of organic solvents for 72 h, with the exception of glycerol, LAC-Yang1 lost nearly all of its activity. Only in the case of glycerol did the enzyme maintain 15.09% of its initial activity.

### 2.3. Degradation of 2,6-Dichlorophenol and 2,3,6-Trichlorophenol by LAC-Yang1

#### 2.3.1. Degradation of 2,6-Dichlorophenol and 2,3,6-Trichlorophenol at Different Concentrations by LAC-Yang1

**Figure 4:**
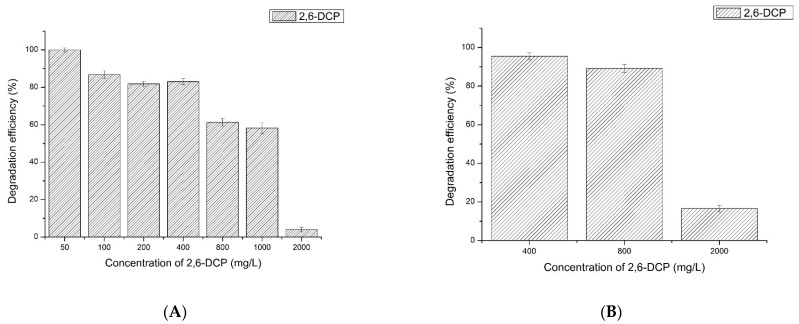

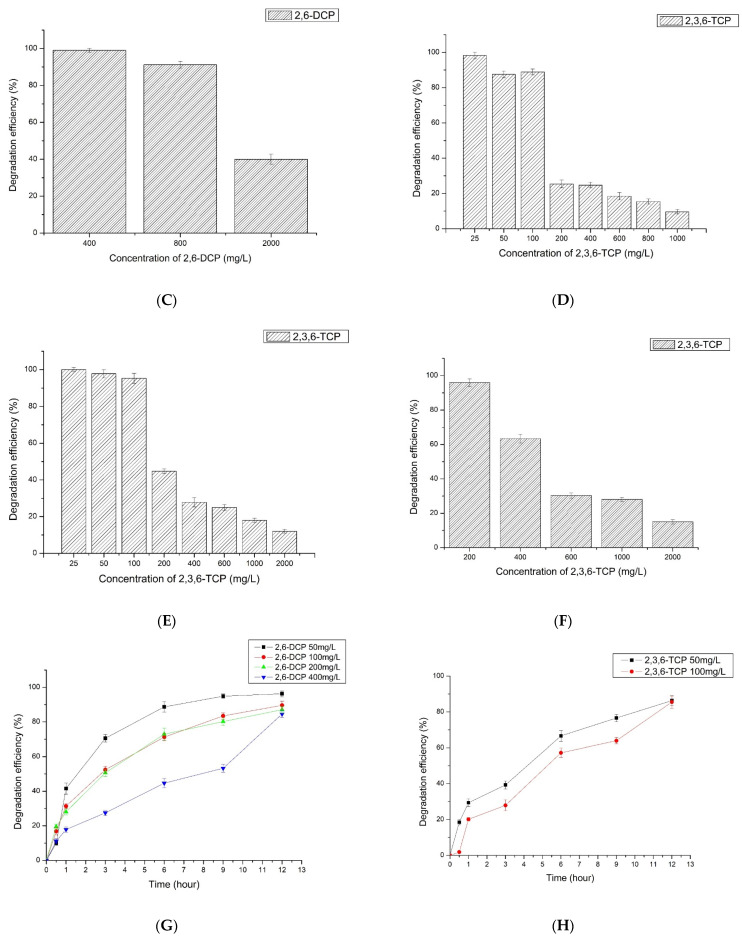

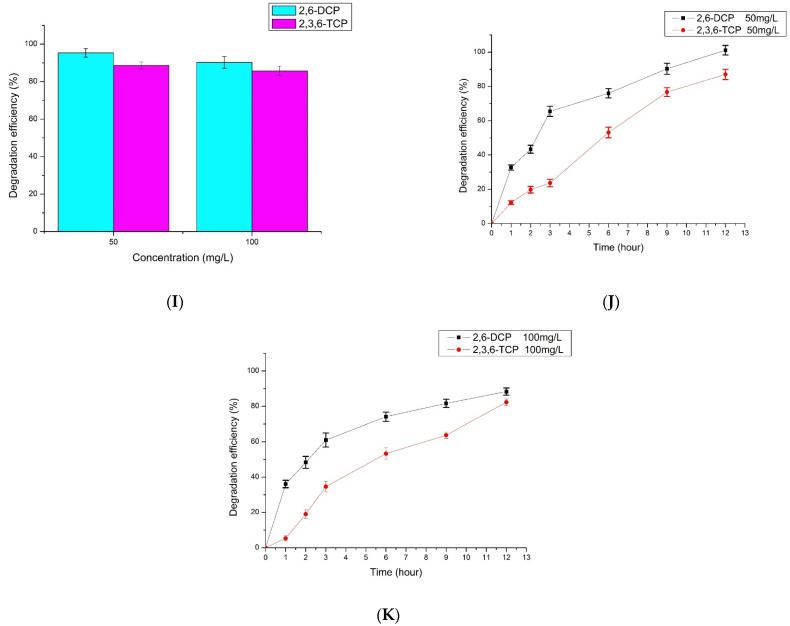
Degradation of 2,6-dichlorophenol (2,6-DCP), 2,3,6-trichlorophenol (2,3,6-TCP) and mixture of 2,6-dichlorophenol and 2,3,6-trichlorophenol by LAC-Yang1. (**A**–**C**): Degradation of different concentrations of 2,6-dichlorophenol by LAC-Yang1 for 12 h. (**A**): LAC-Yang1 (1 U/mL). (**B**): LAC-Yang1 (2 U/mL). (**C**): LAC-Yang1 (4 U/mL). (**D**–**F**): Degradation of different concentrations of 2,3,6-trichlorophenol by LAC-Yang1 for 12 h. (**D**): LAC-Yang1 (1 U/mL). (**E**): LAC-Yang1 (2 U/mL). (**F**): LAC-Yang1 (4 U/mL). (**G**): Time-course analysis of degradation of different concentrations of 2,6-dichlorophenol by LAC-Yang1(1 U/mL). (**H**): Time-course analysis of degradation of different concentrations of 2,3,6-trichlorophenol by LAC-Yang1(1 U/mL). (**I**): Degradation of the mixture of 2,6-dichlorophenol and 2,3,6-trichlorophenol (2,6-DCP + 2,3,6-TCP) by LAC-Yang1. The concentration of 2,6-DCP and 2,3,6-TCP in the mixture was 50 mg/L and 100 mg/L, respectively. (**J**,**K**): Time-course analysis of degradation of the mixture of 2,6-dichlorophenol and 2,3,6-trichlorophenol by LAC-Yang1. (**J**): 2,6-DCP + 2,3,6-TCP, 50 mg/L + 50 mg/L. (**K**): 2,6-DCP + 2,3,6-TCP, 100 mg/L + 100 mg/L.

The degradation of different concentrations of 2,6-dichlorophenol by LAC-Yang1 was studied. As shown in Figure 4A, the 12-h percent degradation of 50 mg/L, 100 mg/L, 200 mg/L, 400 mg/L, 800 mg/L, 1000 mg/L and 2000 mg/L of 2,6-DCP by LAC-Yang1 (1 U/mL) was 100%, 86.70%, 81.85%, 82.98%, 61.35%, 58.23% and 4.10%, respectively. When the laccase activity increased to 4 U/mL, the 12-h percent degradation of 400 mg/L, 800 mg/L, 2000 mg/L of 2,6-DCP increased to 99.10%, 91.20%, 40.11%, respectively (Figure 4C). The above results show the strong degradation ability of LAC-Yang1 for 2,6-DCP, even at higher concentrations of this chlorophenol. The degradation efficiency of LAC-Yang1 toward 2,6-DCP increased with laccase activity.

The degradation of different concentrations of 2,3,6-trichlorophenol by LAC-Yang1 was also studied. As shown in Figure 4D–F, LAC-Yang1 also showed a good degradation ability of 2,3,6-TCP. An increase in laccase activity enhanced its degradation of 2,3,6-TCP. LAC-Yang1 displayed better degradation ability of 2,6-DCP compared to 2,3,6-TCP.

The degradation time course of LAC-Yang1 toward different concentrations of 2,6-DCP and 2,3,6-TCP was further studied. The time-course degradation curve of LAC-Yang1 for different concentrations of 2,6-DCP and 2,3,6-TCP is shown in Figure 4G,H. The enzyme achieved rapid degradation of 2,6-DCP and was able to degrade more than 50% of this substrate within 3 h. The 3-h percent degradation of 50 mg/L and 100 mg/L of 2,3,6-TCP was 39.29% and 27.92%, respectively. The degradation speed of LAC-Yang1 toward 2,3,6-TCP was slower than that toward 2,6-DCP.

#### 2.3.2. Degradation of Chlorophenol Mixtures by LAC-Yang1

The degradation of two chlorophenol mixtures (2,6-DCP + 2,3,6-TCP) by LAC-Yang1 was shown in Figure 4I. When the concentration of both chlorophenols was 100 mg/L in the mixture, the 12-h percent degradation of 2,6-DCP and 2,3,6-TCP by LAC-Yang1 was 90.24% and 85.65%, respectively (Figure 4I). The above results show the strong degradation ability of LAC-Yang1 for both a single type of chlorophenol and for chlorophenol mixtures. The ability of LAC-Yang1 to degrade 2,6-DCP and 2,3,6-TCP in a mixture was not significantly different from the ability to degrade a single chlorophenol. The time-course degradation curve of LAC-Yang1 for the mixture of the two chlorophenols was shown in Figure 4J,K. LAC-Yang1 also achieved a rapid degradation of the chlorophenol mixtures.

#### 2.3.3. Effect of Different Concentrations of Metal Ions on the Degradation of 2,6-Dichlorophenol and 2,3,6-Trichlorophenol by LAC-Yang1: Metal-Ion Tolerance during Degradation

The above results show the good degradation effect of LAC-Yang1 on 2,6-DCP and 2,3,6-TCP. On this basis, metal ions of various concentrations were added to the degradation mixture to investigate their effect on 2,6-DCP and 2,3,6-TCP degradation by LAC-Yang1.

**Figure 5:**
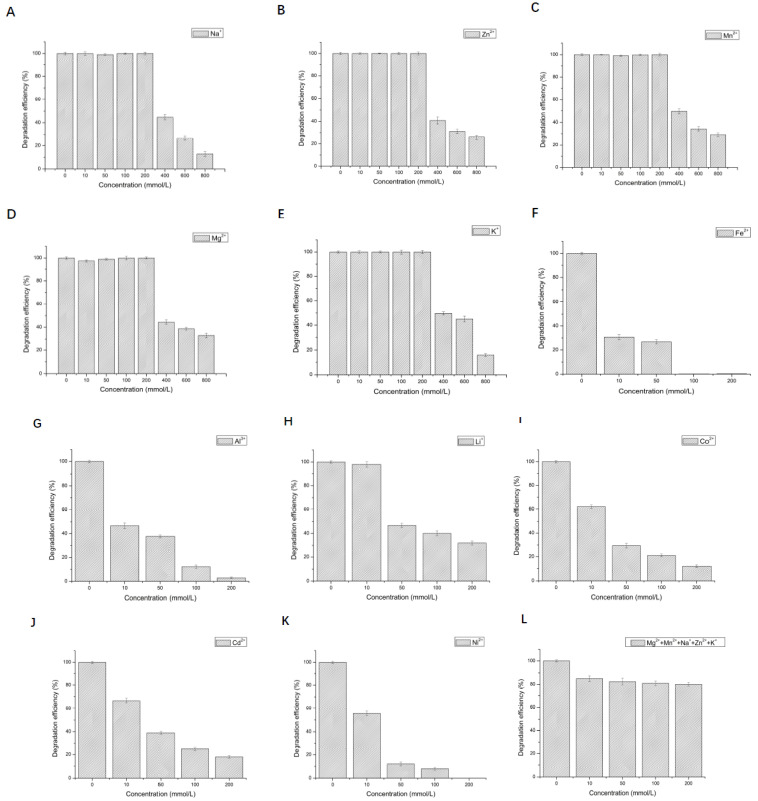

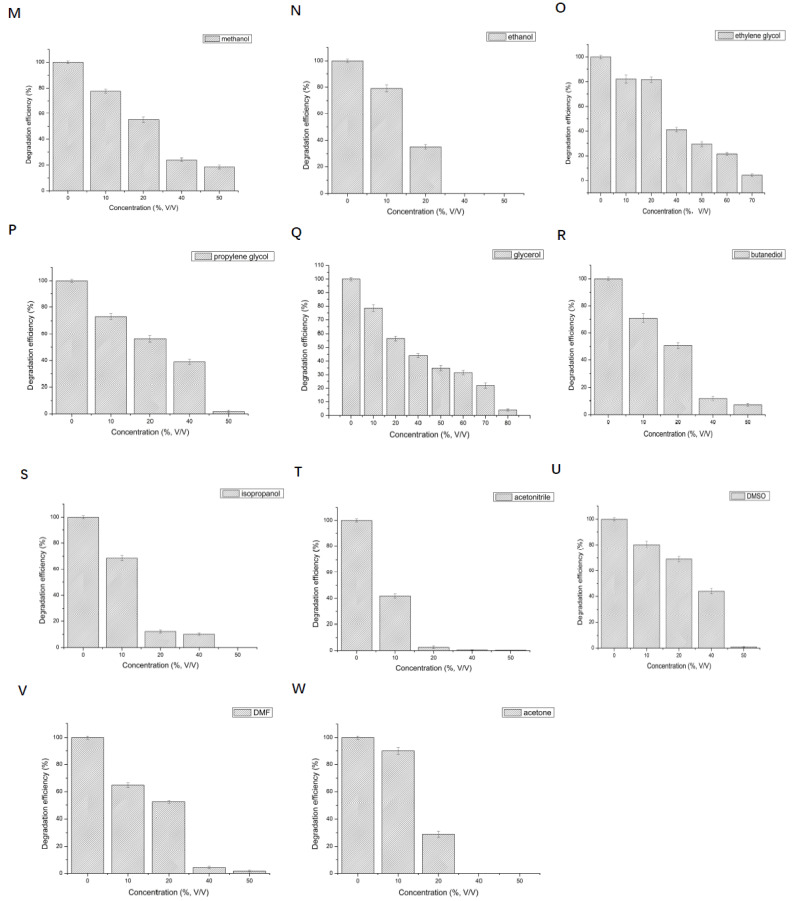
Effect of different metal ions and organic solvents on the degradation of 2,6-dichlorophenol by LAC-Yang1. (**A**): Na^+^, (**B**): Zn^2+^, (**C**): Mn^2+^, (**D**): Mg^2+^, (**E**): K^+^, (**F**): Fe^2+^, (**G**): Al^3+^, (**H**): Li^+^, (**I**): Co^2+^, (**J**): Cd^2+^, (**K**): Ni^2+^, (**L**): mixture of five metal ions (Mg^2+^ + Mn^2+^ + Na^+^ + Zn^2+^ + K^+^). (**M**): methanol, (**N**): ethanol, (**O**): ethylene glycol, (**P**): propylene glycol, (**Q**): glycerol, (**R**): butanediol, (**S**): isopropanol, (**T**): acetonitrile, (**U**): DMSO, (**V**): DMF, (**W**): acetone.

The effects of different concentrations of metal ions on the degradation of 2,6-DCP by LAC-Yang1 were shown in Figure 5A–K. LAC-Yang1 show a strong tolerance to Na^+^, Zn^2+^, Mn^2+,^ Mg^2+^, K^+^ in the degradation of 2,6-DCP. Various metal ions such as Na^+^, Zn^2+^, Mn^2+^, Mg^2+^, K^+^ in the concentration range of 10–200 mM had no obvious effect on the degradation of 2,6-DCP by LAC-Yang1 (Figure 5A–E). LAC-Yang1 could still maintain its degradation ability for the substrate under high concentrations of Na^+^, Zn^2+^, Mn^2+^, Mg^2+^, K^+^ (400–800 mM). As shown in Figure 5F–K, 100 mM of Fe^2+^, Al^3+^, Li^+^, Co^2+^, Cd^2+^ and Ni^2+^ had a strong inhibitory effect on the degradation of 2,6-DCP by LAC-Yang1. Fe^2+^ exerted even stronger inhibition on the degradation (100 mM of Fe^2+^ could completely inhibit the degradation of 2,6-DCP by LAC-Yang1).

**Figure 6:**
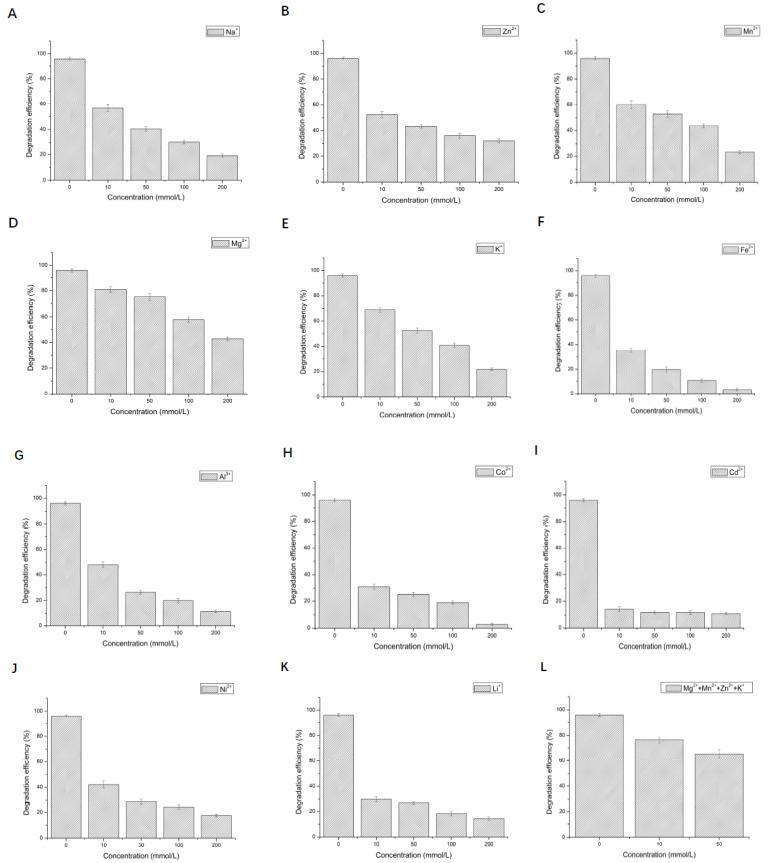

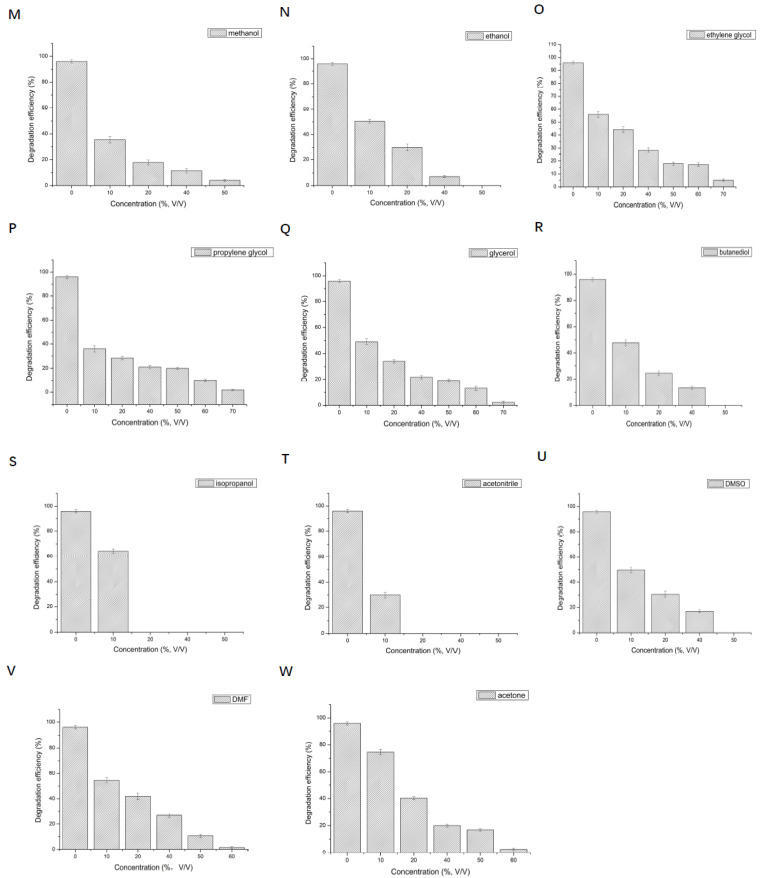
Effect of different metal ions and organic solvents on the degradation of 2,3,6-trichlorophenol by LAC-Yang1. (**A**): Na^+^, (**B**): Zn^2+^, (**C**): Mn^2+^, (**D**): Mg^2+^, (**E**): K^+^, (**F**): Fe^2+^, (**G**): Al^3+^, (**H**): Co^2+^, (**I**): Cd^2+^, (**J**): Ni^2+^, (**K**): Li^+^, (**L**): mixture of four metal ions (Mg^2+^ + Mn^2+^ + Zn^2+^ + K^+^). (**M**): methanol, (**N**): ethanol, (**O**): ethylene glycol, (**P**): propylene glycol, (**Q**): glycerol, (**R**): butanediol, (**S**): isopropanol, (**T**): acetonitrile, (**U**): DMSO, (**V**): DMF, (**W**): acetone.

The effects of different concentrations of metal ions on the degradation of 2,3,6-TCP by LAC-Yang1 were shown in Figure 6A–K. The results indicate the tolerance of this enzyme to Mg^2+^, Mn^2+^, Na^+^, Zn^2+^, K^+^ in its degradation of 2,3,6-TCP, though at a significantly weaker level as compared to 2,6-DCP degradation. Mg^2+^, Mn^2+^, Na^+^, Zn^2+^, K^+^ impacted the LAC-Yang1 degradation of 2,3,6-TCP to varying extent.

The effect of metal ion mixtures on the degradation of 2,6-DCP and 2,3,6-TCP by LAC-Yang1 was further studied. As shown in Figure 5L and Figure 6L, LAC-Yang1 also displayed strong tolerance to metal ion mixtures in its degradation of 2,6-DCP and 2,3,6-TCP.

#### 2.3.4. Effect of Different Concentrations of Organic Solvents on the Degradation of 2,6-Dichlorophenol and 2,3,6-Trichlorophenol by LAC-Yang1: Organic-Solvent Tolerance during Degradation

Following the addition of different concentrations of organic solvents to the degradation mixture, the effects of 11 organic solvents on the degradation of 2,6-DCP and 2,3,6-TCP by LAC-Yang1 were studied.

The effects of different concentrations of organic solvents on the degradation of 2,6-DCP by LAC-Yang1 are shown in Figure 5M–W. LAC-Yang1 shows a strong tolerance to glycerol and ethylene glycol in the degradation of 2,6-DCP. When the concentration of glycerol and ethylene glycol in the system increased to 50% (*v*/*v*), the percent degradation of 2,6-DCP could still reach 34.90% and 29.44%, respectively (Figure 5Q,O), while other organic solvents completely inhibited 2,6-DCP degradation. Among the 11 organic solvents studied, glycerol had the least effect on the degradation of 2,6-DCP by LAC-Yang1, and LAC-Yang1 demonstrated the strongest tolerance toward glycerol.

The effects of different concentrations of organic solvents on the degradation of 2,3,6-TCP by LAC-Yang1 are shown in Figure 6M–W. LAC-Yang1 showed tolerance to a variety of organic solvents, especially ethylene glycol, glycerol, propylene glycol, etc., in its degradation of 2,3,6-TCP. In a comparison of the degradation of 2,6-DCP with that of 2,3,6-TCP, LAC-Yang1 displayed better tolerance to ethylene glycol and glycerol in the case of the former than the latter. Our results also suggest the strong inhibition on LAC-Yang1 degradation of 2,6-DCP and 2,3,6-TCP by acetonitrile and isopropanol (Figure 5T,S and Figure 6T,S).

#### 2.3.5. Toxicity of the Degradation Products of 2,6-Dichlorophenol by LAC-Yang1: Detoxification Effect of the Laccase

**Figure 7:**
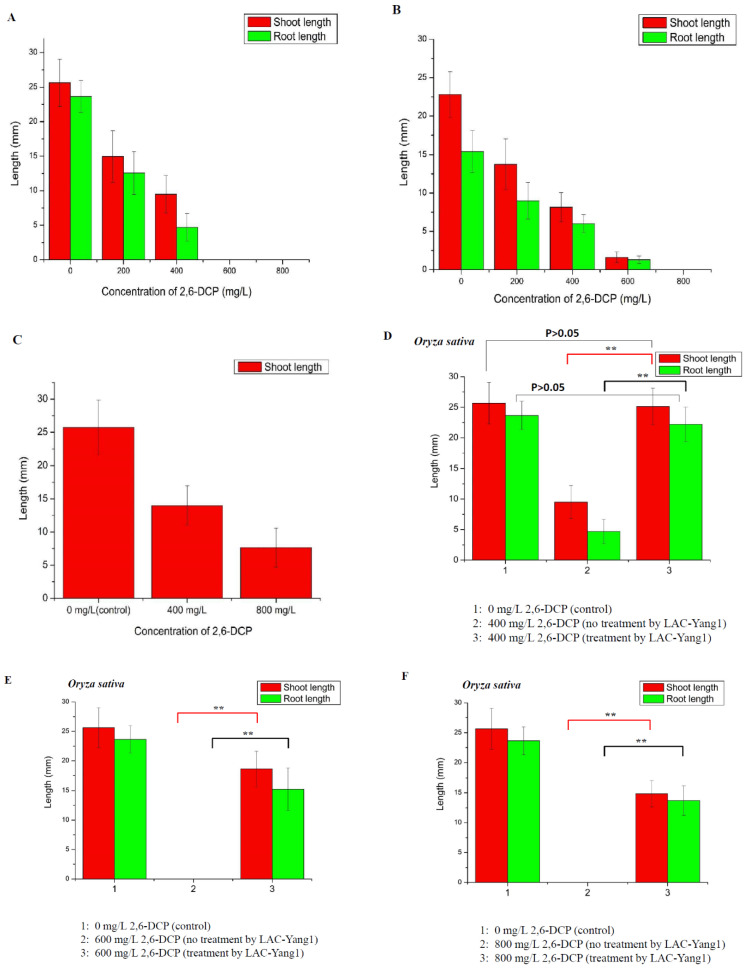

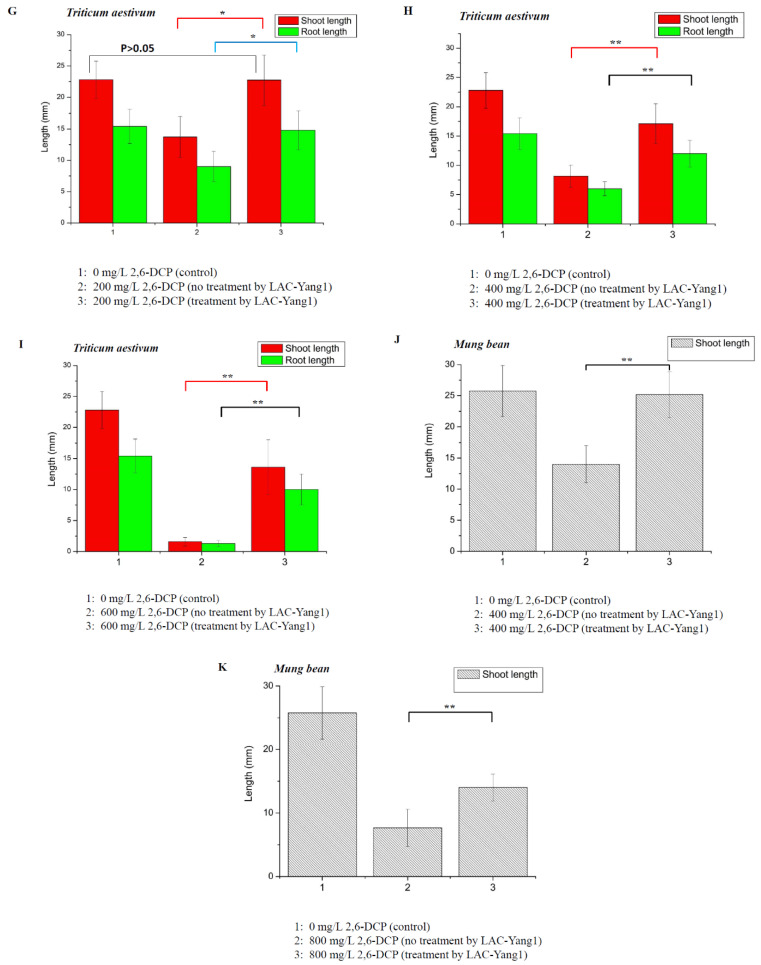
Phytotoxicity study of 2,6-dichlorophenol (2,6-DCP) and its degradation product after treatment by LAC-Yang1. (**A**–**C**)**:** Effect of different concentration of 2,6-DCP on the germination of plant seeds. (**A**): *Oryza sativa*. (**B**): *Triticum aestivum*. (**C**): *Mung bean*. (**D**): Effect of 2,6-DCP (400 mg/L, untreated by LAC-Yang1) and 2,6-DCP (400 mg/L, treated by LAC-Yang1) on the germination of *Oryza sativa* seeds. The seeds treated with distilled water (without 2,6-DCP) was set as the control. (**E**): Effect of 2,6-DCP (600 mg/L, untreated by LAC-Yang1) and 2,6-DCP (600 mg/L, treated by LAC-Yang1) on the germination of *Oryza sativa* seeds. (**F**): Effect of 2,6-DCP (800 mg/L, untreated by LAC-Yang1) and 2,6-DCP (800 mg/L, treated by LAC-Yang1) on the germination of *Oryza sativa* seeds. (**G**): Effect of 2,6-DCP (200 mg/L, untreated by LAC-Yang1) and 2,6-DCP (200 mg/L, treated by LAC-Yang1) on the germination of *Triticum aestivum* seeds. (**H**): Effect of 2,6-DCP (400 mg/L, untreated by LAC-Yang1) and 2,6-DCP (400 mg/L, treated by LAC-Yang1) on the germination of *Triticum aestivum* seeds. (**I**): Effect of 2,6-DCP (600 mg/L, untreated by LAC-Yang1) and 2,6-DCP (600 mg/L, treated by LAC-Yang1) on the germination of *Triticum aestivum* seeds. (**J**): Effect of 2,6-DCP (400 mg/L, untreated by LAC-Yang1) and 2,6-DCP (400 mg/L, treated by LAC-Yang1) on the germination of *Mung bean* seeds. (**K**): Effect of 2,6-DCP (800 mg/L, untreated by LAC-Yang1) and 2,6-DCP (800 mg/L, treated by LAC-Yang1) on the germination of *Mung bean* seeds. *p* < 0.01 indicated that the difference was very significant, which was indicated by **. *p* < 0.05 indicated that the difference was significant, which was indicated by *.

The phytotoxicity of 2,6-DCP before and after its degradation was evaluated by seed germination and growth, by measuring the lengths of shoot and root of germinated seeds (Figure 7).

Firstly, the toxic effects of different concentrations of 2,6-DCP on the germination of three types of plant seeds (*Oryza sativa*, *Triticum aestivum*, *Mung bean*) were measured (Figure 7A–C). As the concentration of 2,6-DCP increased, the germination of rice seeds was clearly inhibited. The length of shoots and roots decreased significantly with the increase in the chlorophenol concentration, such that 600 mg/L of 2,6-DCP completely inhibited the germination of rice seeds. The results demonstrate the strong toxic effect of 2,6-DCP on the germination of rice seeds (Figure 7A). 2,6-DCP also showed strong toxicity to the two other types of plant seeds such as *Triticum aestivum* and *Mung bean* (Figure 7B,C).

Based on the above work, the toxic effect of products resulting from 2,6-DCP degradation by LAC-Yang1 on the germination of the three types of plant seeds was studied (Figure 7D–K). As shown in Figure 7D, 400 mg/L of 2,6-DCP not treated by LAC-Yang1 inhibited the germination of rice seed significantly. However, 400 mg/L of 2,6-DCP treated by LAC-Yang1 had no toxic effect on rice seed germination. There was no significant difference in the length of shoot and root between the group treated with degraded 2,6-DCP (400 mg/L) and the control group (without 2,6-DCP). This result indicated that LAC-Yang1 degradation could completely eliminate the toxic effect of 2,6-DCP (400 mg/L) on rice seed (Figure 7D). As shown in Figure 7E,F, the germination of rice seeds was completely inhibited as the concentration of 2,6-DCP increased to 600 mg/L and 800 mg/L. But the LAC-Yang1-degraded 2,6-DCP had a much weaker toxic effect on rice seed germination than the undegraded 2,6-DCP. Rice seeds treated with degraded 2,6-DCP showed significantly better germination progress than those treated with undegraded 2,6-DCP (the difference was very significant, *p* < 0.01) (Figure 7E,F).

The toxic effects of products resulting from 2,6-DCP degradation by LAC-Yang1 on the germination of the *Triticum aestivum* seed and *Mung bean* seed were shown in Figure 7G–K. For the *Triticum aestivum* seed, there was no significant difference in the length of shoot and root between the group treated with degraded 2,6-DCP (200 mg/L) and the control group (without 2,6-DCP). LAC-Yang1 degradation could completely eliminate the toxic effect of 2,6-DCP (200 mg/L) on wheat seed (Figure 7G). The toxic effects of 400 mg/L and 600 mg/L of 2,6-DCP on the wheat seed germination were significantly decreased after treatment by LAC-Yang1 (Figure 7H,I). For the *Mung bean* seed, LAC-Yang1 degradation could completely eliminate the toxic effect of 2,6-DCP (400 mg/L) on *Mung bean* seed (Figure 7J). The toxic effect of 800 mg/L of 2,6-DCP on the germination of *Mung bean* seed was significantly reduced after treatment by LAC-Yang1 (Figure 7K).

In conclusion, the above results indicate that LAC-Yang1 demonstrated good detoxification effect of 2,6-DCP while degrading this compound. The phytotoxicity of 2,6-DCP after LAC-Yang1 degradation was significantly reduced or eliminated.

## 3. Discussion

### 3.1. Enzymatic Properties of LAC-Yang1

In this study, the laccase LAC-Yang1 was purified from *P. ostreatus* strain yang1, and its enzymatic properties were systematically studied. On this basis, LAC-Yang1 was compared with other reported laccases derived from the genus *Pleurotus* in terms of its enzymatic properties. The enzymatic properties of LAC-Yang1 were compared with other reported laccases in the following aspects: (1) effect of temperature on the activity of laccase, (2) effect of pH on the activity and stability of laccase, (3) effect of inhibitors on the activity of laccase, (4) effect of different metal ions on the activity of laccase, (5) effect of different organic solvents on the activity of laccase, and (6) effect of metal ions and organic solvents on the stability of laccase.

A comparison of the effect of temperature, pH, EDTA and metal ions on the activity of LAC-Yang1 and other reported laccases is summarized in Table 8. As shown in Table 8, the activities of laccases from *P. ostreatus* strain 10969, *P. ostreatus* IBL-02, *P. ostreatus* UAMH7992, *P. ostreatus* UAMH7988, *P. ostreatus* IE-8, *P. ostreatus* strain Florida were reduced to a very low level or were completely inhibited when the temperature exceeded 60 °C [31,32,33,34]. However, in this study, we found that LAC-Yang1 still had catalytic activity at a high temperature. Thus, LAC-Yang1 displayed greater tolerance to high-temperature conditions than other laccases reported previously [31,32,33,34]. Little research has been done on laccase activity at low temperature (<20 °C) in previous studies. LAC-Yang1 remained highly active at low temperature and displayed significantly higher low-temperature activity than the laccase from *Pleurotus nebrodensis* [35]. In summary, compared with other reported laccases from the genus *Pleurotus* [31,32,33,34,35], LAC-Yang1 showed better tolerance to high and low temperature conditions. The high activity level of LAC-Yang1 was maintained across a wide range of temperatures (10–85 °C). This property of LAC-Yang1 is of great value and benefit for its practical application in industry and environmental biotechnology.

In terms of the influence of pH on laccase activity, differences were observed between LAC-Yang1 and other reported laccases derived from the genus *Pleurotus*. As shown in Table 8, the activities of the laccases from *P. ostreatus* strain 10969 [31], *P. ostreatus* 7992 [33], *P. ostreatus* 7972 [33], and *P. ostreatus* 7980 [33] were very sensitive to acidic conditions. However, LAC-Yang1 still maintained some level of activity under extremely acidic conditions. Thus, LAC-Yang1 was more resistant to extreme acidic conditions (pH 1.0–4.0) than the other laccases from *P. ostreatus* strain 10969, *P. ostreatus* 7992, *P. ostreatus* 7972, and *P. ostreatus* 7980 reported previously [31,33].

The stability of LAC-Yang1 at different pH values was compared with other laccases reported previously. Although there have been some reports on the influence of pH on the stability of laccase from the genus *Pleurotus*, most of the previous research was performed at pH 1–7 [31,34,35], with only a few studies on the stability of the enzyme under strong alkaline conditions. Giardina et al. reported that the laccase POXA1b produced by *P. ostreatus* var florida was very stable at alkaline pH. t_1/2_ of POXA1b at pH 9.0 was 30 days [38]. Okamoto et al. found a good stability for the laccase isolated from *P. ostreatus* K16-2 under the alkaline condition of pH 9.0–11.0. The residual activity of the laccase from *P. ostreatus* K16-2 was about 90% after incubation at pH 9.0 for 20 h. However, the previous study only detected residual laccase activity after 20 h of incubation at pH 9.0, 10.0, and 11.0 and no stability after a longer duration of incubation (such as 24 h or more) under strong alkaline conditions. The stability of the laccase from *P. ostreatus* K16-2 after incubation at pH 9.0–11.0 for a longer time (such as 72 h and 7 days) was not studied in the previous research [37]. Our results also show the good stability of LAC-Yang1 under highly alkaline conditions. However, different from the previous research [37], the stability of LAC-Yang1 after incubation at pH 9.0–11.0 for a longer time (up to 7 days) was investigated in our study. LAC-Yang1 still possessed 71.57%, 67.58%, and 64.50% of its original activity after long-term incubation (7 days) at pH 9, 10, and 11, respectively.

In terms of the effect of inhibitors on laccase activity, LAC-Yang1 showed strong resistance to some enzyme inhibitors such as EDTA and SDS, which was the significant advantage of this laccase. Comparison of the effect of EDTA on the activity of LAC-Yang1 and other reported laccases was summarized in Table 8. The results show the superior resilience of LAC-Yang1 to EDTA compared with the laccases from *Trametes polyzona* [38], *P. ostreatus* K16-2 [37], and *P. ostreatus* strain 10969 [31]. The resistance of LAC-Yang1 to EDTA was significantly stronger than that of other laccases reported previously [31,37,38].

In terms of the influence of different metal ions on laccase activity, we also compared the metal-ion tolerance of LAC-Yang1 with that of other laccases reported previously. As shown in Table 8, LAC-Yang1 showed stronger tolerance to Mg^2+^, Mn^2+^, Zn^2+^, and Cu^2+^ than the laccase from *P. ostreatus* strain 10969 [31]. Compared with the laccase from *Trametes polyzona* KU-RNW027 [38], LAC-Yang1 showed a stronger resistance to Na^+^ and Co^2+^. Taken together, compared with other laccases reported previously [31,38], LAC-Yang1 exhibited stronger tolerance and resistance to different metal ions such as Mg^2+^, Mn^2+^, Zn^2+^, Cu^2+^, Na^+^, and Co^2+^. LAC-Yang1 had good broad-spectrum tolerance to various metal ions.

While many studies have examined the effect of metal ions on laccase activity, fewer studies have examined the effect of organic solvents on laccase activity. However, previous research did not examine the effects of glycerol, propylene glycol, ethylene glycol, and butanediol on laccase activity [31,33,38,39]. In this study, LAC-Yang1 was found to have strong tolerance to glycerol and propylene glycol. Among 11 organic solvents, LAC-Yang1 was most resistant to glycerol.

To our knowledge, there are few reports on laccase stability in the presence of different metal ions and organic solvents. The effect of different metal ions and organic solvents on the stability of laccase from *P. ostreatus* strain 10969, *Trametes polyzona* KU-RNW027, *Pycnoporus* sp. SYBC-L10, *Trametes maxima* IIPLC-32, *P. ostreatus* 7988, and *P. ostreatus* 7980 was not investigated in previous research [14,31,33,37,38,39]. In this study, in addition to the effects of metal ions and organic solvents on laccase activity, their effects on laccase stability were also systematically studied. Our results show the good stability of LAC-Yang1 in the presence of Mg^2+^, Mn^2+^, glycerol and ethylene glycol. Such findings were not reported in previous research [14,31,33,37,38,39]. In this study, we found that the activity of LAC-Yang1 decreased even after short time in the presence of glycerol and propylene glycol. The possible reason for this phenomenon is explained as follows. Hydrophobic force and hydrogen bond play an important role in maintaining the natural structure and stability of enzyme protein [40,41]. Previous studies have indicated that organic solvents such as glycerol can denature and inactivate enzyme protein by destroying hydrophobic forces and hydrogen bonds. Organic solvents can be used as denaturants to inactivate proteins, but different organic solvents have different denaturing capabilities [42,43]. Glycerol and propylene glycol as organic solvents may cause partial denaturation and inactivation of the laccase by affecting the hydrogen bonds and hydrophobic forces, which may lead to the reduction of laccase activity. However, previous studies have shown that the ability of glycerol to denature enzyme proteins is relatively weak [42]. Magsumov et al. reported that glycerol had the weakest ability to denature lysozyme [42]. Therefore, compared with other organic solvents used in this study, the stability of LAC-Yang1 laccase in glycerol is the highest. Among the 11 organic solvents studied, LAC-Yang1 is most stable in the presence of glycerol.

In summary, compared to other laccases reported previously, LAC-Yang1 has the following novelty and advantages in terms of enzymatic properties: (1) LAC-Yang1 displayed greater tolerance to high-temperature conditions than some other laccases. LAC-Yang1 also remained highly active at low temperature. The high activity level of LAC-Yang1 could be maintained across a wide range of temperatures (10–85 °C). (2) LAC-Yang1 showed a strong tolerance to extremely acidic conditions and strong stability under strong alkaline conditions. (3) LAC-Yang1 displayed stronger tolerance and resistance to different inhibitors such as EDTA and SDS. The resistance of LAC-Yang1 to EDTA was significantly stronger than that of some other laccases reported previously. (4) LAC-Yang1 exhibited stronger tolerance to different metal ions such as Mg^2+^, Mn^2+^, Zn^2+^, Cu^2+^, Na^+^, and Co^2+^ than some other laccases reported previously. (5) LAC-Yang1 had strong tolerance toward some organic solvents such as glycerol and propylene glycol. LAC-Yang1 was most resistant to glycerol. (6) LAC-Yang1 showed good stability in the presence of Mg^2+^, Mn^2+^, glycerol and ethylene glycol. The above characteristics and advantages of LAC-Yang1 give this enzyme great value and potential in industrial applications.

### 3.2. Degradation and Detoxification of Chlorophenol by LAC-Yang1

Chlorophenols are chlorine-containing aromatic compounds which can be directly used in modern industry. Chlorophenols are widely used as insecticides, herbicides, wood preservatives and acaricides. In addition, chlorophenols are widely used in the production of various chemical products as important raw chemical materials. 2,6-dichlorophenol is one of the important raw materials for the synthesis of non-steroidal antipyretic analgesics and herbicides. Chlorophenol is also a key intermediate compound in the industrial production of pesticides, dyes, phenolic resins and paper [17]. The extensive use of chlorophenol has led to its release into the environment mainly through the following ways: (1) In the process of directly using chlorophenols as pesticides, herbicides and wood preservatives, residual chlorophenols will be released into the environment. (2) Chlorophenols are used as chemical raw materials for the manufacture and production of various chemical products, and are released into soil or water with the discharge of industrial wastewater. Industrial wastewater containing a large amount of chlorophenol will be produced and discharged in the process of chemical production. Taken together, the massive discharge of industrial wastes and industrial wastewater containing various chlorophenols has led to the release of chlorophenols into the environment (soil, water, etc.). The heavy use of chlorophenols in modern industry (used directly, used as raw material and intermediate compound) has caused them to be discharged into the natural environment through various channels, seriously damaging the agricultural environment and human health [17,44,45]. In addition, previous research has suggested that chlorophenols released into the environment also come from the degradation of some contaminants. Previous studies have shown that various herbicides and insecticides, especially 2,4-dichlorophenoxyacetic acid (2,4-D) and 2,4,5-trichlorophenoxyacetic acid (2,4,5-T), can be degraded by microorganisms to produce a large number of chlorophenol intermediate metabolites, which can be further decomposed into 2,4-dichlorophenol and 2,4,5-trichlorophenol [44]. The use of chlorine-containing bleaching agents in the pulp and paper industry will produce a large amount of chlorophenol compounds such as chlorinated lignin. Chlorine bleaching wastewater from pulp and paper industry also contains a large number of chlorophenols with different structures [46]. Although chlorophenols have a wide range of uses in industrial production and human activities, they are persistent, stubborn and highly toxic compounds that are widely present in the environment and water bodies, showing high acute toxicity and genotoxicity [18,44,47,48]. Chlorophenols entering the human body will damage the human nervous system and respiratory system, causing endocrine disorders. Chlorophenols also have potential carcinogenic effects [49,50]. Therefore, an in-depth study on the degradation of chlorophenols is very important for eliminating the damage caused by chlorophenol pollutants to the environment and human health.

In this study, LAC-Yang1 had a strong ability to degrade and detoxify chlorophenols. Compared to the previous studies on the degradation of chlorophenol by other laccases [20,21,22,23,24,25,26,27,28], LAC-Yang1 has the following novelty and advantages in terms of chlorophenol degradation: (1) There is no report on the tolerance of purified laccase to different metal ions and organic solvents in the degradation of chlorophenol in previous research [20,21,22,23,24,25,26,27,28]. In this study, the tolerance of laccase to different metal ions and organic solvents during the degradation of chlorophenols was systematically explored for the first time. The strong and broad-spectrum tolerance of LAC-Yang1 to various metal ions and organic solvents in the degradation of chlorophenols is one of the main novelties of our study. This remarkable feature suggests that LAC-Yang1 has great practical value and prospect in the treatment of chlorophenol-polluted wastewater containing different metal ions and organic solvents. LAC-Yang1 has good application potential in the efficient treatment of actual chlorophenol pollutants of complex composition encountered in real life. (2) Previous studies on chlorophenol degradation by laccase mostly focused on the ability of laccase to degrade chlorophenols [20,21,22,23,24,25,26,27]. However, the detoxification effect on chlorophenol by purified laccase was not reported in the above-mentioned studies [20,21,22,23,24,25,26,27]. Very little research on the detoxification of chlorophenol by laccase has been performed [28,51]. There is no report on the detoxification effect of laccase on the phytotoxicity of chlorophenol. The phytotoxicity of chlorophenol before and after degradation by laccase was not investigated in previous research [28,51]. In this study, the phytotoxicity of chlorophenol (2,6-DCP) before and after laccase treatment was evaluated for the first time. The strong ability of LAC-Yang1 to reduce or even eliminate the phytotoxicity of chlorophenol is also a major innovation in this study.

### 3.3. The Perspectives of Use of LAC-Yang1 Laccase: In Which Areas and Performing What

Because of its low substrate specificity, a wide range of substrates and unique degradation function, laccase is widely used in the fields of industrial and environmental biotechnology. Our study suggests that LAC-Yang1 laccase showed strong ability to degrade and detoxify chlorophenols. Compared to other laccases reported previously, LAC-Yang1 had unique and novel characteristics in terms of enzymatic properties. Therefore, LAC-Yang1 has a good application prospect and value in the field of environmental biotechnology and bioremediation, such as the efficient degradation of persistent organic pollutants that are difficult to degrade. The perspectives of use of LAC-Yang1 laccase are described as follows. In the area of environmental pollutant degradation, LAC-Yang1 laccase can be used in the following aspects: (1) Wastewater polluted by chlorophenols usually contains a large number of metal ions and organic solvents besides chlorophenol. In the treatment of chlorophenol wastewater from different sources and with complex composition, these metal ions and organic solvents may affect the ability of laccase to degrade chlorophenols. Our study indicates that LAC-Yang1 had strong ability to degrade high concentrations of chlorophenols and chlorophenol mixtures. LAC-Yang1 also showed a strong tolerance toward a variety of metal ions and organic solvents in its degradation of chlorophenols. Therefore, LAC-Yang1 can be used in the treatment of actual industrial wastewater containing high concentrations of chlorophenols in the future. LAC-Yang1 can be practically applied to the effective treatment and degradation of chlorophenol-polluted wastewater containing different metal salts and organic solvents. LAC-Yang1 laccase has great potential to achieve efficient degradation and detoxification of chlorophenol pollutants in the actual polluted environment (actual chlorophenol pollutants and contaminated wastewater). (2) LAC-Yang1 laccase may be used for the degradation and detoxification of other environmental organic pollutants except chlorophenols, such as polycyclic aromatic hydrocarbons (PAHs, fluorene, phenanthrene, fluoranthene, pyrene, etc.), industrial dyes (azo dye, anthraquinone dye, indigo dye, etc.), dyeing wastewater, environmental endocrine disruptors, and pesticides. Our other study suggested that LAC-Yang1 had a strong ability to degrade polycyclic aromatic hydrocarbons such as fluorene. (3) In order to better improve the reusability and stability of LAC-Yang1 in the degradation of chlorophenol pollutants, laccase needs to be immobilized. Immobilized laccase can often achieve good reusability. Therefore, we will further study the degradation and detoxification of different chlorophenols and industrial wastewater containing chlorophenols by immobilized laccase. LAC-Yang1 laccase can be immobilized on different media, and the reusability of immobilized LAC-Yang1 in the degradation of chlorophenols may be greatly improved.

## 4. Materials and Methods

### 4.1. Strain and Culture Conditions

*P. ostreatus* strain yang1 was preserved in Central China Normal University, Wuhan, China. The tomato juice medium was used for the production of laccase. After incubation at 28 °C in a shaking incubator (180 rpm) for 3 days, syringic acid and Cu^2+^ were added to the culture. After addition of the inducers, the fungal cultures were then grown at 28 °C with continuous shaking at 180 rpm. Laccase activity was measured daily. The fungal cultures were harvested at the peak of laccase activity. The laccase activity was measured as described previously using 2,2′-azino-bis-(3-ethylbenzothiazoline-6-sulfonic acid) (ABTS) as the substrate [52].

### 4.2. Purification of Laccase Named as LAC-Yang1 from P. ostreatus Strain Yang1

Fungal cultures at the peak of laccase activity were collected and centrifuged at 5000× *g* for 20 min. The culture supernatant was precipitated by (NH_4_)_2_SO_4_ (80% saturation) at 4 °C overnight. The precipitates were dissolved in 20 mmol/L citric acid-disodium hydrogen phosphate dodecahydrate buffer (CPBS, pH 6.5) and dialyzed in a dialysis bag (MW: 8000–14,000) for 48 h to remove ammonium sulfate. The dialyzed crude enzyme was then concentrated using PEG20,000, filtered, and applied to the DEAE Sepharose anion-exchange chromatography column equilibrated with 20 mmol/L CPBS (pH 6.5). The laccase protein was eluted with a linear gradient of 0–1 mol/L NaCl in the 20 mmol/L CPBS (pH 6.5) at a 1 mL/min flow rate. Proteins in the eluted fractions were monitored by measuring the absorbance at 280 nm. The activity of laccase was measured. The active fractions were collected and dialyzed by 20 mM CPBS (pH 6.5) for removing NaCl. The dialyzed enzyme fractions were subsequently applied to the Phenyl-6FF (HS) hydrophobic chromatography column equilibrated with 20 mM CPBS (pH 6.5) containing 1 mol/L (NH_4_)_2_SO_4_. The laccase protein was eluted with a linear gradient of 1–0 mol/L (NH_4_)_2_SO_4_ in the same buffer at a 1 mL/min flow rate. Fractions possessing laccase activity were collected, dialyzed, concentrated and stored at 4 °C. The purity of the purified laccase was detected by SDS-PAGE which contained a 5% polyacrylamide-stacking gel and a 12% polyacrylamide-separating gel. The purified laccase was named as LAC-Yang1. Native-PAGE (non-denaturing polyacrylamide gel electrophoresis) was also used to detect LAC-Yang1 laccase. The main step of Native-PAGE is described as follows. (1) Preparation of protein electrophoresis gel (gel preparation): The gel for Native-PAGE contained a 5% polyacrylamide-stacking gel and a 12% polyacrylamide-separating gel. Different from SDS-PAGE, 10% SDS was not added in the preparation of gel for Native-PAGE. (2) Sample loading and electrophoresis: The original activity of the protein needs to be maintained in Native-PAGE. Therefore, the protein sample was not subjected to denaturation treatment such as boiling, and the sample was directly mixed with the non-denatured protein loading buffer and loaded. (3) Stain of gel (colouration): The laccase substrate 2,6-Dimethylphenol (2,6-DMP) was used as the staining agent to detect the active laccase. After electrophoresis, the protein gel was peeled off and washed twice with distilled water. Then, the gel was stained with the acetic acid-sodium acetic buffer (pH 5.0) containing 1.0 mM 2,6-DMP. The laccase protein with laccase activity could oxidize 2,6-DMP and show a brownish-yellow band on the protein band. The active laccase protein could react specifically with 2,6-DMP and show a brownish-yellow band on the protein band.

### 4.3. Kinetic Studies on the Purified LAC-Yang1

Kinetic studies were performed in 50 mM acetic acid-sodium acetic buffer (pH 5.0) at 30 °C. ABTS and 2,6-DMP (both 0.01, 0.02, 0.04, 0.06, 0.08 and 0.1 mM) and guaiacol (0.1, 0.2, 0.4, 0.6, 0.8 and 1 mM) were used as substrates. The absorbance at 420 nm, 470 nm and 465 nm was measured respectively. The amounts of laccase used for each substrate were the same (0.01 U/mL). The kinetic parameters of LAC-Yang1 were calculated by non-linear regression model based on the Michaelis–Menten equation.

### 4.4. Effect of pH and Temperature on the Activity of Purified LAC-Yang1

The effect of different pH on the activity of LAC-Yang1 was determined in citric acid-disodium hydrogen phosphate dodecahydrate buffer (50 mM) within a pH range of 1.0–8.0 at 30 °C using ABTS (1 mM) as the substrate. The amount of the laccase was 0.01 U/mL. The laccase activity measured at the optimal pH was set as 100%.

The effect of different temperatures on the activity of LAC-Yang1 was determined in acetic acid-sodium acetic buffer (50 mM, pH 5.0) within a temperature range of 10–85 °C using ABTS (1 mM) as the substrate. The amount of the laccase was 0.01 U/mL. The laccase activity measured at the optimal temperature was set as 100%.

### 4.5. Effect of pH and Temperature on the Stability of Purified LAC-Yang1

To evaluate the pH stability, the purified LAC-Yang1 was incubated at different pH (1–12) at 30 °C for 3, 6, 12, 72 and 168 h, respectively. The buffer used was citric acid-disodium hydrogen phosphate dodecahydrate buffer (50 mM). The amount of the laccase was 0.1 U/mL. The laccase activity was measured at different time points using ABTS (1 mM) as the substrate. Then, the residual laccase activity was calculated based on the original activity before incubation. The initial activity of LAC-Yang1 was set as 100%.

To evaluate the thermal stability, the purified LAC-Yang1 was incubated at 30–70 °C for 1, 2, 3, 6, 9, 12 and 24 h, respectively. The buffer used was acetic acid–sodium acetic buffer (50 mM). The amount of the laccase was 0.1 U/mL. The laccase activity was measured at different time points using ABTS (1 mM) as the substrate. Then, the residual laccase activity was calculated based on the original activity before incubation. The initial activity of LAC-Yang1 was set as 100%.

### 4.6. Effect of Inhibitors on the Activity of Purified LAC-Yang1

The effects of different concentrations of EDTA-2Na (0.5, 5, 10, 25, 50, 100, 200 and 400 mM), SDS (100, 200 and 400 mM), mercaptoethanol (0.005, 0.05 and 0.5 mM), sodium azide (0.0005, 0.005 and 0.05 mM), and DTT (0.0005, 0.005 and 0.05 mM) on the laccase activity were measured in 50 mM acetic acid–sodium acetic buffer (pH 5.0) containing different inhibitors at 30 °C. The amount of the laccase was 0.01 U/mL. An amount of 1 mM ABTS was used as the substrate. The residual activity was calculated based on the control in the absence of any inhibitor (set as 100%).

### 4.7. Effect of Metal Ions on the Activity and Stability of Purified LAC-Yang1

The effects of different metal ions including K^+^, Na^+^, Mg^2+^, Mn^2+^, Cu^2+^, Zn^2+^, Al^3+^, Fe^2+^, Cd^2+^, Co^2+^, Li^+^, Ni^2+^ (final concentration: 10–800 mM) on the laccase activity of LAC-Yang1 were determined by measuring the residual activity relative to the control without adding any metal compound. The laccase activity of the control was set as 100%. The buffer used to evaluate the effect of metal ions on the activity of laccase was 50 mM acetic acid–sodium acetic buffer (pH 5.0). The amount of the laccase was 0.01 U/mL. 1 mM ABTS was used as the substrate for measuring the laccase activity.

To evaluate the effect of different metal ions on the stability of LAC-Yang1, the purified LAC-Yang1 was incubated with different concentrations of metal ions (2, 5 and 50 mM) at 30 °C for 7 days. Then the laccase activity was measured at different time points (0, 3, 6, 12, 24, 72 and 168 h). The residual activity was calculated based on the original activity before incubation. The initial activity of LAC-Yang1 was set as 100%. The buffer used to evaluate the effect of metal ions on the stability of laccase was 50 mM acetic acid–sodium acetic buffer (pH 5.0). The amount of the laccase was 0.1 U/mL. 1 mM ABTS was used as the substrate for measuring the laccase activity.

### 4.8. Effect of Organic Solvents on the Activity and Stability of Purified LAC-Yang1

The effects of different organic solvents including methanol, ethanol, ethylene glycol, propylene glycol, glycerol, isopropanol, butanediol, acetonitrile, acetone, DMSO, DMF (final concentration: 5%–70%, *v*/*v*) on the laccase activity of LAC-Yang1 were determined by measuring the residual activity relative to the control without adding any organic solvent. The laccase activity of the control was set as 100%. The buffer used to evaluate the effect of organic solvents on the activity of laccase was 50 mM acetic acid–sodium acetic buffer (pH 5.0). The amount of the laccase was 0.01 U/mL. 1 mM ABTS was used as the substrate for measuring the laccase activity.

To evaluate the effect of different organic solvents on the stability of LAC-Yang1, the purified LAC-Yang1 was incubated with different concentrations of organic solvents (5%, 10%, 50%, *v*/*v*) at 30 °C for 7 days. Then, the laccase activity was measured at different time points (0, 3, 6, 12, 24, 72 and 168 h). The residual activity was calculated based on the original activity before incubation. The initial activity of LAC-Yang1 was set as 100%. The buffer used to evaluate the effect of organic solvents on the stability of laccase was 50 mM acetic acid-sodium acetic buffer (pH 5.0). The amount of the laccase was 0.1 U/mL. 1 mM ABTS was used as the substrate for measuring the laccase activity.

### 4.9. Degradation of 2,6-Dichlorophenol and 2,3,6-Trichlorophenol by LAC-Yang1

The reaction mixture contained (final concentration): 25–2000 mg/L chlorophenol (2,6-dichlorophenol, 2,3,6-trichlorophenol, dissolved in methanol), 50 mM acetic acid-sodium acetic buffer (pH 5.0), purified LAC-Yang1 (the amount of the laccase was 1 U/mL, 2 U/mL and 4 U/mL). The total volume of the reaction mixture was 2 mL. Degradation was performed at 30 °C for 12 h. The reaction mixture was then extracted with an equal volume of ethyl acetate. A Shimadzu LC-20A HPLC (Shimadzu, Kyoto, Japan) equipped with a diode array detector was used to measure the concentration of 2,6-dichlorophenol and 2,3,6-trichlorophenol. The chromatographic grade methanol was used as the mobile phase. An amount of 20 µL of each sample was injected into the HPLC. Chromatographic separation was performed using a WondaSil C18 column (250 mm × 4.6 mm × 5 µm) at a flow rate of 1.0 mL/min at room temperature. The method used for elution was linear gradient elution (60–90% methanol). 2,6-dichlorophenol and 2,3,6-trichlorophenol were detected at 215 nm.

The degradation efficiency of chlorophenol was expressed as a degradation percentage (%) which was calculated by the following formula: Degradation efficiency (%) = (C_0_ − C_t_)/C_0_ × 100, where C_0_ was the initial concentration of chlorophenol before degradation and C_t_ was the residual concentration at time t.

Degradation of the mixture of 2,6-dichlorophenol and 2,3,6-trichlorophenol (2,6-DCP + 2,3,6-TCP) by LAC-Yang1 was also performed as described above. 2,6-dichlorophenol and 2,3,6-trichlorophenol were added into the reaction mixture together (2,6-DCP + 2,3,6-TCP, 50 mg/L + 50 mg/L; 2,6-DCP + 2,3,6-TCP, 100 mg/L + 100 mg/L).

### 4.10. Effects of Metal Ions and Organic Solvents on the Degradation of 2,6-Dichlorophenol and 2,3,6-Trichlorophenol by LAC-Yang1

To evaluate the effects of metal ions and organic solvents on the degradation of chlorophenol by LAC-Yang1, different concentrations of metal ions and organic solvents were added into the reaction mixture, respectively. The reaction mixture contained (final concentration): 50 mg/L 2,6-dichlorophenol or 2,3,6-trichlorophenol, 50 mM acetic acid-sodium acetic buffer (pH 5.0), purified LAC-Yang1 (the amount of the laccase was 1 U/mL), different metal ions (10 mM–800 mM) or different organic solvents (10–70%, *v*/*v*). Degradation was performed at 30 °C for 12 h. The reaction mixture without adding metal ions and organic solvents was set as the control. Degradation was monitored and calculated as described above.

### 4.11. Phytotoxicity Study of 2,6-Dichlorophenol before and after the Degradation by LAC-Yang1

The phytotoxicity of 2,6-dichlorophenol before and after its degradation was evaluated by plant seed germination and growth. The toxic effects of different concentrations of 2,6-dichlorophenol (not treated or treated by LAC-Yang1) on the germination of three types of plant seeds (*Oryza sativa*, *Triticum aestivum* and *Mung bean*) were measured according to the method described before [53]. The phytotoxicity study was conducted (at room temperature) in relation to *Oryza sativa*, *Triticum aestivum* and *Mung bean* (20 seeds of each) by watering separately 10 mL of original 2,6-dichlorophenol (200 mg/L, 400 mg/L, 600 mg/L, 800 mg/L) and 2,6-dichlorophenol treated by LAC-Yang1. Plant seeds were also treated with distilled water at the same time as the control. The shoot and root lengths of *Oryza sativa*, *Triticum aestivum*, *Mung bean* treated under different conditions were measured after 5 days, 3 days, 2 days, respectively.

### 4.12. Statistical Analysis

The data were presented as mean ± standard error. Statistical analyses were performed using GraphPad Prism 5 software (GraphPad Software, San Diego, CA, USA). A *t* test was used to analyze the difference between the control group and the test group. *p*-value < 0.01 indicated very significant difference (presented as **). *p*-value < 0.05 indicated significant difference (presented as *).

## 5. Conclusions

A laccase LAC-Yang1 was successfully purified from a white-rot fungus strain *P. ostreatus* strain yang1 with high laccase activity. The enzymatic properties of LAC-Yang1 and its ability to degrade and detoxify chlorophenols were systematically studied. LAC-Yang1 possesses unique and novel characteristics in terms of enzymatic properties as well as chlorophenol degradation and detoxification compared to other laccases from other fungi reported previously. LAC-Yang1 showed a strong tolerance to extremely acidic conditions and strong stability under strong alkaline conditions. LAC-Yang1 exhibited strong tolerance to different inhibitors, metal ions and organic solvents. LAC-Yang1 also had a good stability in the presence of some metal ions and organic solvents. Our results show the strong degradation ability of LAC-Yang1 for 2,6-dichlorophenol, 2,3,6-trichlorophenol and chlorophenol mixtures (2,6-dichlorophenol + 2,3,6-trichlorophenol). Our study provides new laccase resources for the efficient degradation and detoxification of chlorophenol pollutants.

## Figures and Tables

**Table 1:** Purification of LAC-Yang1 laccase from *P. ostreatus* strain yang1.

Purification Steps	Total Activity (U)	Total Protein (mg)	Specific Activity (U/mg)	Purified Fold	Yield (%)
crude enzyme	22,424.67	501.6	44.81	1	100
precipitation with ammonium sulfate	20,602.48	172.34	119.55	2.67	99.87
anion exchange chromatography	1528.70	21.14	72.32	1.61	6.82
hydrophobic chromatography	384.93	6.42	63.64	0.35	1.72

**Table 2:** The kinetic parameters of purified LAC-Yang1.

	V_max_ (×10^−7^ mM/s)	K_m_ (mM)	K_cat_ (s^−1)^	K_cat_/K_m_ (s^−1^ mM^−1^)
ABTS	4.41 ± 0.22	0.15 ± 0.05	73.48 ± 1.15	503.29
2,6-DMP	0.60 ± 0.14	0.12 ± 0.03	9.98 ± 1.10	80.48
guaiacol	0.35 ± 0.12	0.81 ± 0.15	5.90 ± 0.70	7.26

**Table 3:** Effect of different temperature and pH on the stability of LAC-Yang1. t_1/2_: the time required for laccase activity to decrease to 50% of the initial enzyme activity. The initial activity of LAC-Yang1 was set as 100%.

Temperature or pH	t_1/2_ (h)
30 °C	6
40 °C	3
50 °C	<1
60 °C	<1
pH 1.0	<3
pH 2.0	<3
pH 3.0	<3
pH 4.0	<3
pH 5.0	<3
pH 6.0	<3
pH 7.0	<3
pH 8.0	<3
pH 9.0	>168
pH 10.0	>168
pH 11.0	>168

**Table 4:** Effect of pH on the stability of LAC-Yang1. The initial activity of LAC-Yang1 was set as 100%.

pH	Residue Activity after 3 h (%)	Residue Activity after 24 h (%)	Residue Activity after 168 h (%)
1.0	2.03	0.89	0.69
2.0	34.38	5.04	0
3.0	30.14	1.58	0.8
4.0	24.14	1.19	0
5.0	31.08	4.54	0
6.0	34.15	16.22	3.87
7.0	36.86	14.13	2.34
8.0	37.82	22.25	7.19
9.0	99.24	93.35	71.56
10.0	96.51	86.16	67.58
11.0	93.76	77.69	64.50

**Table 5:** Effect of different inhibitors on the activity of purified LAC-Yang1. The laccase activity of the control without adding any inhibitor was set as 100%.

Inhibitor	Concentration (mM)	Relative Activity (%)
SDS	100	92.17
	200	91.39
	400	84.73
EDTA-2Na	0.5	98.18
	5	96.66
	10	94.81
	25	87.10
	50	81.16
DTT	0.0005	44.42
	0.005	36.70
	0.05	0.48
Sodium azide	0.0005	98.44
	0.005	52.24
	0.05	8.01
Mercaptoethanol	0.005	88.21
	0.05	75.27
	0.5	4.37

**Table 6:** Effect of different metal ions on the stability of LAC-Yang1. t_1/2_: the time required for laccase activity to decrease to 50% of the initial enzyme activity. The initial activity of LAC-Yang1 was set as 100%. The final concentration of metal ions was 2 and 5 mM.

Metal Ions (Concentration)	t_1/2_ (h)
K_2_SO_4_ (2 mM)	3
Na_2_SO_4_ (2 mM)	3
MgSO_4_ (2 mM)	24
Al_2_(SO_4_)_3_ (2 mM)	3
ZnSO_4_ (2 mM)	6
CuSO_4_ (2 mM)	3
MnSO_4_ (2 mM)	12
K_2_SO_4_ (5 mM)	3
Na_2_SO_4_ (5 mM)	3
MgSO_4_ (5 mM)	12
Al_2_(SO_4_)_3_ (5 mM)	3
ZnSO_4_ (5 mM)	6
CuSO_4_ (5 mM)	3
MnSO_4_ (5 mM)	12

**Table 7:** Effect of different organic solvents on the stability of LAC-Yang1. t_1/2_: the time required for laccase activity to decrease to 50% of the initial enzyme activity. The initial activity of LAC-Yang1 was set as 100%. The final concentration of organic solvents was 5% and 10% (*v*/*v*).

Organic Solvents	t_1/2_ (h)
ethylene glycol (5%)	24
glycerol (5%)	72
ethanol (5%)	12
methanol (5%)	3
butanediol (5%)	12
propylene glycol (5%)	12
acetonitrile (5%)	6
isopropanol (5%)	12
acetone (5%)	12
DMSO (5%)	12
DMF (5%)	12
ethylene glycol (10%)	12
glycerol (10%)	24
ethanol (10%)	6
methanol (10%)	3
butanediol (10%)	6
propylene glycol (10%)	3
acetonitrile (10%)	3
isopropanol (10%)	6
acetone (10%)	6
DMSO (10%)	6
DMF (10%)	6

**Table 8:** Comparison of the enzymatic properties of LAC-Yang1 with other reported laccases. Effect of temperature, pH, EDTA and metal ions on the activity of LAC-Yang1 and other reported laccases.

Laccase	Relative Activity (%) at Different Temperature	Relative Activity (%) at Different pH	Relative Activity (%) at a Certain Concentration of EDTA	Relative Activity (%) at Different Metal ions	Ref.
laccase from *Pleurotus ostreatus* strain 10969	<30% at 80 °C	10% at pH 2.0	40% at 10 mM of EDTA	60% at 5 mM of Mg^2+^	[31]
		0 at pH 1.0		60% at 5 mM of Mn^2+^	
				60% at 5 mM of Zn^2+^	
				60% at 5 mM of Cu^2+^	
laccase from *Pleurotus ostreatus* IBL-02	0 at 70 °C				[32]
laccases from *Pleurotus ostreatus* UAMH7992	0 at 60 °C	0 at pH 4.0			[33]
laccases from *Pleurotus ostreatus* IE-8	0 at 60 °C				[33]
laccases from *Pleurotus ostreatus* UAMH7988	0 at 60 °C				[33]
laccase from *Pleurotus ostreatus* strain Florida	<20% at 65 °C				[34]
laccase from *Pleurotus nebrodensis*	20% at 10 °C				[35]
	40% at 20 °C				
laccases from *Pleurotus ostreatus* UAMH7972		<20% at pH 4.0			[33]
laccases from *Pleurotus ostreatus* UAMH7980		<20% at pH 4.0			[33]
laccase from *Pleurotus ostreatus* K16-2			55% at 1 mM of EDTA		[36]
laccase from *Trametes polyzona* KU-RNW027			0 at 50 mM of EDTA	65.5% at 10 mM of Na^+^	[37]
				0 at 1 mM of Co^2+^	
LAC-Yang1	85.47% at 60 °C 83.50% at 65 °C 56.44% at 80 °C 34.72% at 85 °C 48.30% at 10 °C 64.20% at 20 °C	87.61% at pH 2.0 25.81% at pH 1.0 76.57% at pH 4.0	96.66% at 5 mM of EDTA 94.81% at 10 mM of EDTA 87.10% at 25 mM of EDTA 81.16% at 50 mM of EDTA		This study
				98.18% at 10 mM of Mg^2+^ 95.58% at 10 mM of Mn^2+^ 97.90% at 10 mM of Zn^2+^ 98.80% at 10 mM of Cu^2+^ 99.0% at 10 mM of Na^+^ 59.04% at 10 mM of Co^2+^ 38.50% at 50 mM of Co^2+^	

## Data Availability

The data presented in this study are available on request from the corresponding author.

## Supplementary Materials

The following are available online. Figure S1: Native-PAGE analysis of the crude laccase produced by *Pleurotus ostreatus* strain yang1. Lane 1–3: the crude laccase solution from *P. ostreatus* strain yang1.

## Sample Availability

Samples of the compounds are not available from the authors.
