# Supplementary material for: Characterization of a Novel Laccase LAC-Yang1 from White-Rot Fungus Pleurotus ostreatus Strain Yang1 with a Strong Ability to Degrade and Detoxify Chlorophenols
Source: Molecules. 2021 Jan 18;26(2):473. doi: 10.3390/molecules26020473 (PMC7829762; doi:10.3390/molecules26020473)

Fig.S1 Native-PAGE analysis of the crude laccase produced by *Pleurotus ostreatus* strain yang1. Lane 1-3: the crude laccase solution from *P. ostreatus* strain yang1.

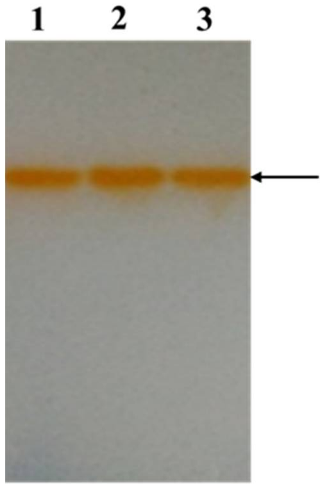

Supplement: Supplementary file 1 [file molecules-26-00473-s001.pdf]
